# Human and Canine Seropositivity to *Toxocara canis* in Uralsk, Kazakhstan: Canine Egg Shedding and Evaluation of an Experimental ELISA

**DOI:** 10.3390/pathogens15080837

**Published:** 2026-08-12

**Authors:** Timur Davlyatshin, Aidar Namet, Mukhit Orynbayev, Kulyaisan Sultankulova, Sabyrkhan Barmak, Asylzhan Myrzakhmet, Oralkhan Tusipkanuly, Dulat Inkarbekov, Bayzhan Omarov, Aiganym Tussipova, Berik Khairullin

**Affiliations:** 1 Scientific Research and Production Center «MVA GROUP» LLP, 1124, Accounting Quarter 096, Koksay Village, Irgeli Rural District, Karasay District, Almaty 040921, Almaty Region, Kazakhstan; 2 LLP «Research Institute for Biological Safety Problems» JSC «QazBioPharm» of the Ministry of Health of the Republic of Kazakhstan, Gvardeysky 080409, Zhambyl Region, Kazakhstan; 3 Republican State Enterprise on the Right of Economic Management «Institute of Zoology», Committee of Science, Ministry of Science and Higher Education of the Republic of Kazakhstan, 93 Al-Farabi Avenue, Almaty 050060, Almaty Region, Kazakhstan

**Keywords:** *Toxocara canis*, toxocariasis, One Health, IgG avidity, stray dogs, copromicroscopy, ELISA, Kazakhstan

## Abstract

Background: The aim of the present study was to develop and optimize an experimental ELISA test system for the detection of IgG antibodies to *Toxocara canis* and subsequently apply it for the assessment of human and canine exposure in Uralsk, Kazakhstan, within a One Health framework. Methods: A native somatic antigen of *T. canis* was fractionated by gel filtration, and the working fraction was selected by SDS-PAGE and pilot ELISA. The optimized test system was used for the analysis of sera of 1000 adult volunteers. Antibody avidity was determined in IgG-positive human specimens, and human seropositivity was evaluated for associations with demographic and questionnaire-derived variables. Serum samples from 100 stray dogs were tested by ELISA and fecal samples by copromicroscopy. Agreement with the commercial DRG ELISA was tested with 50 positive and 50 negative human serum samples. Results: The antigen fraction of 18–22 kDa showed the best separation between positive and negative sera. IgG antibodies against *T. canis* were detected in 116/1000 volunteers (11.6%). Seropositivity was 17.0% in the 2025 sample and 6.2% in the 2026 sample (odds ratio 3.10; 95% confidence interval 2.01–4.77; *p* < 0.001). No significant associations were found with sex, age, residence, or questionnaire-derived variables. Among seropositive volunteers, 28.4% had low-avidity, 26.7% intermediate-avidity, 29.3% intermediate/high-avidity and 15.5% high-avidity IgG. Overall, 78/100 of the stray dogs were copropositive, and 93/100 were seropositive. All copropositive dogs were seropositive, and 15 additional seropositive dogs were copronegative. Agreement with the DRG ELISA was 97.0% (Cohen’s κ = 0.94). Conclusions: The experimental ELISA allowed the assessment of *T. canis* exposure of humans and dogs. Seropositivity in humans in Uralsk showed exposure. High seropositivity in dogs and frequent egg shedding showed a substantial canine reservoir and a potential for environmental contamination. These results support integrated surveillance and control within a One Health context.

## 1. Introduction

Toxocariasis is a zoonotic helminth infection mainly caused by the larvae of *Toxocara canis* and *Toxocara cati*. Dogs and other canids are the definitive hosts of *T. canis*, and cats and other felids are the definitive hosts of *T. cati*. The adult worms live in the small intestine and lay eggs that are passed out in the stool. The eggs embryonate and become infective in the environment. Toxocara spp. does not require an intermediate host, but mammalian and avian paratenic hosts are possible in which the larvae remain in the tissues but do not develop into adult worms. Definitive hosts are infected by ingestion of embryonated eggs or infected paratenic hosts. *T. canis* can also be transmitted from female dogs to their offspring, especially by transplacental transmission [1,2,3].

Humans are accidental paratenic hosts and are infected mainly by ingestion of embryonated eggs in contaminated soil, food, or water or by contaminated hands. Infection can also occur by ingesting raw or undercooked tissues of paratenic hosts. In humans, the larvae migrate through various tissues but do not develop into adult worms. The intensity of the disease depends on the number of larvae, the organs involved, and the immune response of the host. Infection may be asymptomatic or cause visceral, ocular, neurological, covert, or common toxocariasis. Eggs are not found in human feces, and larvae are rarely detected directly because adult worms do not mature in humans. Diagnosis is therefore mainly based on specific antibodies and clinical and laboratory findings. Anti-Toxocara IgG reflects exposure but is not sufficient to confirm active disease on its own, whereas IgG avidity provides further information about the antibody response [2,4,5].

Toxocariasis is a neglected disease with global distribution [6]. The global seroprevalence of anti-Toxocara is estimated to be 19.0% but varies widely between regions and study populations [7]. The number of free-roaming animals, access to veterinary care, deworming practices, sanitation and environmental contamination all influence human exposure. Dogs with intestinal infection shed eggs that can survive in soil and contaminate food, water, hands and the environment of the house. Human serology, canine serology, and canine copromicroscopy therefore provide complementary information on human exposure, canine exposure, and egg shedding [8,9,10].

The aims of this study were: (1) to evaluate the capacity of an experimental IgG ELISA based on a native somatic antigen of *T. canis* to discriminate between positive and negative sera and to determine its agreement with a commercial ELISA; (2) to determine IgG seropositivity and avidity profiles among adult volunteers and to assess associations with demographic and questionnaire data; and (3) to determine IgG seropositivity and egg shedding among stray dogs and to compare the canine serological and copromicroscopic findings. The combined analyses were used to assess the human exposure and canine reservoir of *T. canis* in Uralsk according to a One Health concept.

## 2. Materials and Methods

### 2.1. Study Design and Setting

We conducted a cross-sectional study of humans and dogs in Uralsk, West Kazakhstan Region, Kazakhstan, in 2025–2026. Samples of human serum were tested for IgG antibodies against *T. canis*. IgG avidity testing was performed on seropositive samples. Serum and fecal samples of stray dogs were evaluated by serology and copromicroscopy, respectively.

An experimental enzyme-linked immunosorbent assay (ELISA) based on a native somatic antigen of *T. canis* was designed and optimized before testing the study samples. The agreement with the commercial DRG *Toxocara canis* IgG ELISA (DRG Instruments GmbH, Marburg, Germany; Cat. No. EIA-3518) was studied with a selected panel of human serum samples.

### 2.2. Human Study Population, Recruitment, and Eligibility

Adult volunteers were recruited in Uralsk (Oral; ca. 51.23° N, 51.37° E), during 2025–2026. Recruitment was conducted through consecutive enrollment: all eligible individuals who attended the participating collection sites and agreed to take part were enrolled until 500 participants had been included in each study year, giving a total sample of 1000 participants.

Before any study procedure, each potential participant received information about the study and signed written informed consent. Participants then completed the volunteer questionnaire and provided a venous blood sample.

The inclusion criteria were age ≥ 18 years, residence in the study area, signed informed consent, agreement to complete the questionnaire and provide a blood sample, no anthelmintic treatment during the previous three months, and availability of a suitable serum sample and complete questionnaire. The exclusion criteria were age < 18 years, inability or refusal to provide informed consent, contraindication to venipuncture, anthelmintic treatment during the previous three months, withdrawal from the study, an incomplete questionnaire, or an unsuitable serum sample. No restrictions were applied based on sex.

### 2.3. Questionnaire and Contextual Variables

After providing informed consent, participants completed a volunteer questionnaire covering sex, age, place of residence, household conditions, contact with soil and domestic animals, access to a garden or land plot, drinking-water sources, fishing and hunting, and selected dietary and food-handling practices.

Age was recorded in years and grouped as 18–29, 30–44, 45–59, 60–74, and ≥75 years. Questionnaire data were evaluated for associations with human IgG seropositivity. Each participant was assigned a unique four-digit study code, which was used to link questionnaire and laboratory data without including personal identifiers in the analytical dataset.

### 2.4. Human Blood Collection and Serum Handling

Venous blood (5–7 mL) was collected by qualified medical personnel into disposable vacuum tubes containing a clot activator. After clotting for 20–30 min, the samples were centrifuged at 1500–2000× *g* for 10 min. Serum was transferred into coded tubes and divided into aliquots of at least 0.5 mL when sufficient volume was available.

Serum aliquots were maintained at 2–8 °C during transport to the laboratory and stored at −20 °C until testing. Samples with insufficient volume, marked hemolysis or lipemia, or visible contamination were excluded. Before analysis, the required aliquot was thawed and brought to room temperature. Samples were tested individually and were not pooled.

### 2.5. Stray Dog Population and Specimen Collection

Specialists of the Zhardem-Vet Training, Research, and Production Center of the Zhangir Khan West Kazakhstan Agrarian and Technical University examined stray canines. The stray dogs of Uralsk were individually examined by a veterinarian. Sex was recorded and age estimated on the basis of dental traits and total physical maturation. Dogs were grouped by estimated age as follows: <1 year of age, 1–3 years of age, and >3–6 years of age. A venous blood specimen and a fresh fecal specimen were collected from each dog and labeled with the same individual animal identifier. Blood was collected in disposable tubes, allowed to clot, and centrifuged at 1500–2000× *g* for 10 min. The serum was separated, aliquoted, and stored at −20 °C until analysis of IgG. Fecal samples were collected in sterile, leak-proof containers separately and transported to the laboratory and examined by copromicroscopy. The samples were labeled with respective animal identification codes and transported to MVA GROUP LLP for further serological and copromicroscopic examinations.

### 2.6. Copromicroscopic Examination of Canine Feces

The canine feces were examined by the Fülleborn flotation technique [11]. A quantity of fecal matter (about 3–5 g) was homogenized in saturated sodium chloride solution and filtered to eliminate gritty particles. After flotation, the surface preparation was examined by light microscopy (BIOBASE Group, Jinan, Shandong, China) at ×100 and ×400 magnification. *Toxocara canis* ova were identified by their specific size, almost spherical shape, strong shell, and rough surface. A sample was considered positive if at least one morphologically suitable *T. canis* egg was detected. Copromicroscopic results were related to serological results in dogs by individual animal identification numbers.

### 2.7. Preparation of Native Somatic Antigen

Adult *T. canis* worms were provided by the Republican State Enterprise on the Right of Economic Management “Institute of Zoology” of the Committee of Science, Ministry of Science and Higher Education of the Republic of Kazakhstan. The worms had been recovered from the small intestine of naturally infected wild jackals during postmortem parasitological examination. No experimentally infected animals or laboratory-maintained parasite cycles were used.

Species identification was based on morphological examination of the adult worms using body shape, cervical alae, and caudal and reproductive structures. Only specimens identified as *T. canis* and morphologically distinct from Toxascaris leonina were used. Molecular confirmation was not performed.

The worms were washed several times with chilled buffer and frozen at ≤−24 °C. Frozen material was homogenized (BIOBASE Group, Jinan, Shandong, China) on ice and extracted with chilled phosphate-buffered saline or low-salt Tris buffer containing EDTA at 500 mg/L. The parasite material-to-buffer ratio was 1:5–1:10. Extraction was performed with gentle mixing on ice for 30–60 min. The homogenate was centrifuged (BIOBASE Group, Jinan, Shandong, China) at 15,000× *g* for 15 min at 4 °C, and the soluble supernatant was collected as the native somatic antigen extract.

### 2.8. Antigen Fractionation and Pilot ELISA

The native somatic antigen extract was separated by gel filtration. The molecular-weight profiles of the collected fractions were examined by sodium dodecyl sulfate–polyacrylamide gel electrophoresis (SDS-PAGE) (Bio-Rad Laboratories, Hercules, CA, USA).

The fractions were screened by pilot indirect ELISA. An equal amount of protein from each fraction was adsorbed onto 96-well polystyrene plates, while the serum dilution, conjugate dilution, incubation, washing, and substrate-development conditions were kept constant. Human serum samples showing different levels of ELISA reactivity were used for comparison. Blank optical density, serum optical density, background signal, and the ratio between high- and low-reactivity samples were calculated for each fraction. The fraction taken forward for assay development was selected based on high signal separation and low background.

### 2.9. Optimization of ELISA Parameters

Optimization was performed in a series of comparative ELISA runs. Standard/medium-binding and high-binding microplates were evaluated. Antigen loads of 20, 40, 50, 75, 100, and 150 µg per plate were compared.

The effects of no blocking and blocking with 0.5% bovine serum albumin, 1% casein, or 0.5% fish gelatin were assessed. Anti-human IgG-HRP (Sigma-Aldrich, St. Louis, MO, USA) conjugate was tested at dilutions of 1:5000, 1:10,000, 1:20,000, 1:30,000, 1:50,000, and 1:100,000. The goat anti-canine IgG-POD conjugate (XEMA, Moscow, Russia) was evaluated separately at dilutions of 1:5000, 1:10,000, 1:20,000, and 1:30,000.

Serum and conjugate incubation periods of 15, 20, 25, 30, and 35 min at 37 °C and 25, 30, and 45 min at room temperature were compared. TMB (Thermo Fisher Scientific, Waltham, MA, USA) development times of 10, 15, and 20 min at 20–25 °C were assessed.

For every tested condition, blank and serum optical-density values were recorded, and signal separation was calculated. Conditions were compared based on signal separation, background optical density, and repeatability. Intra-assay repeatability was assessed using repeated measurements within the same run, while inter-assay reproducibility was evaluated in three independent runs performed on different days. Mean optical density, standard deviation, and coefficient of variation were calculated.

### 2.10. Human and Canine Serological Analysis

All 1000 human serum samples and 100 canine serum samples were examined using the experimental indirect ELISA under the conditions established during optimization. The selected native somatic antigen fraction was adsorbed onto microplates, followed by the addition of diluted serum samples. After incubation and washing, anti-human IgG–HRP conjugate was added to human samples, whereas goat anti-canine IgG-POD (peroxidase) conjugate was used for canine samples.

After conjugate incubation, the plates were washed, and TMB substrate was added. The reaction was stopped with an acidic stop solution. Optical density was measured at 450 nm with a reference wavelength of 620–650 nm. All samples were tested in duplicate.

### 2.11. Quality Control and Interpretation of ELISA

Each analytical run included blank wells and the assay controls required to monitor background and reagent activity. A run was accepted when the background signal and assay-control values met the established criteria and when the coefficient of variation between duplicate measurements was <15%. The mean optical density of the duplicate wells was used for interpretation.

The cut-off was calculated separately for each analytical run:

OD_cut-off_ = OD_NC_ + 0.200

where OD_NC_ is the mean optical density of the negative assay control.

The positivity index was calculated as

PI = OD_sample_/OD_cut-off_

Samples with PI ≥ 1.0 were classified as positive, whereas samples with PI < 1.0 were classified as negative.

Western blot testing was not performed, and clinical symptoms and eosinophil counts were not assessed. Human IgG-positive results were therefore interpreted as evidence of exposure to *T. canis* and not as confirmation of active clinical toxocariasis.

### 2.12. Determination of Human IgG Avidity

The avidity of anti-*T. canis* IgG was determined in all seropositive human samples using a paired-well urea ELISA based on a previously described method [12]. Each sample was tested in parallel in two antigen-coated wells: one without denaturing treatment and one treated with urea. Urea concentrations of 4.0, 6.0, and 8.0 M were evaluated during optimization. The final assay used 6.0 M urea for 10 min at 20–25 °C. After treatment, the ELISA was completed using the same anti-human IgG–HRP conjugate, substrate, and optical-density measurement conditions as the standard human IgG assay.

The avidity index (AI) was calculated as follows:

AI (%) = (OD_urea_/OD_without urea_) × 100

where OD_urea_ is the optical density after urea treatment and OD_without urea_ is the optical density of the corresponding untreated well. The avidity profiles were classified according to the criteria presented in Table 1.

### 2.13. Comparison to Commercial Assay

After testing all 1000 human serum samples with the experimental ELISA, a balanced panel of 100 samples was selected for comparison with the commercial assay. The panel included 50 samples classified as positive and 50 classified as negative by the experimental ELISA.

Samples were eligible for inclusion when the duplicate ELISA result met the quality-control criteria, the sample had a clear positive or negative classification, sufficient serum volume remained for additional testing, the identification code and storage history were complete, and there was no marked hemolysis, lipemia, or visible contamination. Selection was not based on demographic or questionnaire data.

Separate aliquots of the selected samples were tested using the DRG *Toxocara canis* IgG ELISA (DRG Instruments GmbH, Marburg, Germany; Cat. No. EIA-3518) according to the manufacturer’s instructions. Results were cross-tabulated as positive or negative for each assay. Overall percentage agreement, positive percentage agreement, negative percentage agreement, and Cohen’s κ were calculated. Because neither assay was treated as an independent reference standard, the comparison was reported as agreement rather than diagnostic sensitivity and specificity.

### 2.14. Statistical Analysis

Statistical evaluation was conducted utilizing GraphPad Prism, version 11.0 (GraphPad Software, Boston, MA, USA). For categorical variables, counts and percentages are used, together with the proportion and 95% confidence interval. The human anti-Toxocara IgG results were analyzed for the entire sample and stratified by year of study, gender, age cohort, and urban or rural living conditions. Relationships between categorical variables were assessed using Pearson’s χ^2^ test or Fisher’s exact test for low expected cell counts. Odds ratios with 95% confidence intervals were calculated for 2 × 2 comparisons.

Canine serological and copromicroscopic results were arranged in a paired 2 × 2 table for each individual animal. The agreement between the two doggy techniques was summarized as observed agreement and Cohen’s kappa. The difference between paired proportions of positivity was evaluated by McNemar’s test. Dog characteristics were categorized by sex and estimated age group.

The MVA GROUP–DRG comparison was done by calculating the positive percentage agreement, negative percentage agreement, overall agreement, and Cohen’s kappa [13] from the 2 × 2 table. The McNemar’s test was used for the analysis of incongruent paired outcomes. Human avidity indices were pooled as continuous measures and grouped into previously defined avidity groupings. All assessments were bilateral. A *p*-value of less than 0.05 was considered statistically significant.

### 2.15. Acceptance of Ethical Considerations

Human protocols were in accordance with the Declaration of Helsinki [14]. All animal procedures were performed following the approved protocol, European Convention for the Protection of Vertebrate Animals used for Experimental and Other Scientific Purposes (Strasbourg, 1986) and WHO Operational Guidelines for Ethics Committees that Evaluate Biomedical Research (Geneva, 2000) [15,16].

The protocol was approved by the Bioethics Committee of the Research and Production Center of MVA GROUP LLP (Protocol No. 1, 8 January 2025) and the Ethics Committee of the Association of Bioethics and Medical Law (Meeting No. 20, 8 January 2025). All participants involved gave written informed consent before recruitment. The data of the study were analyzed using different codes.

## 3. Results

### 3.1. Antigen Fraction Selection and ELISA Optimization

Gel-filtration fractions of the native somatic *T. canis* antigen were examined by SDS-PAGE and pilot ELISA. The 18–22 kDa fraction provided the clearest separation, with optical densities of 1.881 and 0.055 for positive and negative reactions, respectively, and a positive-to-negative ratio of 34.20.

The final configuration of the experimental ELISA is presented in Table 2. The same antigen-coated plate format and general assay procedure were used for human and canine sera. However, the conjugates were optimized separately for the two host species. Anti-human IgG–HRP was used for human sera, while goat anti-canine IgG-POD IgG–POD was used for canine sera.

The intra- and inter-assay coefficients of variation remained below the predefined acceptance limit (Table 3).

### 3.2. Human IgG Seropositivity

IgG antibodies to *T. canis* were detected in 116 of 1000 participants, giving an overall seropositivity of 11.6% (95% CI: 9.8–13.7%). The samples enrolled in 2025 and 2026 differed significantly in seropositivity (OR = 3.10; 95% CI: 2.01–4.77; *p* < 0.001). No significant associations were found with sex, age group, residence, or questionnaire variables (Table 4).

### 3.3. Avidity of Human Anti-Toxocara IgG

All 116 seropositive human samples underwent avidity testing. The distribution of avidity profiles is presented in Table 5. An avidity index of ≥60% was found in 52 samples (44.8%).

### 3.4. Agreement with the Commercial DRG ELISA

In total, 3 of the 100 serum samples produced discordant results between the experimental and commercial assays (Table 6). Overall percentage agreement was 97.0% (95% CI: 91.5–99.0%); positive percentage agreement was 94.0% (95% CI: 83.8–97.9%); and negative percentage agreement was 100.0% (95% CI: 92.9–100.0%). Cohen’s κ was 0.94, and the exact McNemar test was not significant (*p* = 0.250).

### 3.5. Findings in Stray Dogs

Serological and copromicroscopic findings according to sex and estimated age are summarized in Table 7.

The paired distribution is presented in Table 8. Overall agreement between serology and copromicroscopy was 85.0% (95% CI: 76.7–90.7%), with Cohen’s κ = 0.42. The difference between paired positive proportions was significant by the exact McNemar test (*p* < 0.001).

## 4. Discussion

### 4.1. Main Findings and One Health Analysis

This study combined human serology and IgG avidity with canine serology and copromicroscopy in Uralsk. These measurements describe different parts of the *T. canis* transmission cycle. Human IgG indicates previous or current exposure; canine IgG indicates exposure of the definitive host; and detection of eggs in dog feces indicates intestinal infection with active egg shedding. Considered together, the findings show human exposure alongside a substantial canine reservoir, supporting a One Health interpretation [17,18,19,20,21].

Dogs are central to the transmission cycle because they are the definitive hosts in which adult *T. canis* worms mature and produce eggs. However, environmental contamination was not measured directly in this study. Canine egg shedding therefore indicates the potential for contamination but does not demonstrate the presence, distribution, viability, or infectivity of eggs in soil or water.

### 4.2. Human Seropositivity in the National and International Context

The human seropositivity of 11.6% was similar to the 11% reported in a rural community in eastern Kazakhstan [22]. It was lower than the global estimate of 19.0% but close to the estimate of 10.5% for the WHO European Region [7]. Considerable variation has been reported across Europe because of differences in study populations, climate, socioeconomic conditions, antigen preparations, cut-off values, and validation procedures [23,24]. In Russia, adult seroprevalence ranged from 3% in Yakutia to 36% in Rostov-on-Don, with an average of approximately 20% across five study populations [25]. The present findings therefore fall within the broad range reported for the region.

Seropositivity was higher in the 2025 than in the 2026 cohort. However, the cohorts had different participants, and anti-*T. canis* IgG may persist after exposure. Therefore, the observed difference in transmission cannot be seen as a change from one year to another and can be accounted for by differences in cohort composition, environmental exposure, hygiene, geophagia, dog ownership, veterinary care, deworming practices or other unmeasured factors.

Seropositivity was not significantly associated with sex, age, residence or questionnaire variables. Lack of statistical associations does not rule out the effect of behavioral and environmental exposures. IgG antibodies may be a marker of previous exposure or exposure outside the current residential setting, while questionnaire data are subject to the variables included and the accuracy of recall by participants. More-consistent reports are associated with direct animal contact in children. Sanitation, soil contact, and contaminated water may be more important in some populations [26,27,28].

IgG seropositivity alone does not confirm active clinical toxocariasis. Diagnosis requires interpretation of serology together with clinical findings, exposure history, and supporting laboratory information. Antibodies may persist; asymptomatic seropositivity is common; and cross-reactivity with other helminths may occur [19,29,30,31,32,33,34]. Accordingly, the human findings in this study represent serological evidence of exposure rather than the prevalence of clinical disease.

The avidity results showed different stages of IgG maturation among seropositive participants. However, avidity cannot determine the exact time of infection and has no direct role in treatment decisions. Previous studies suggest that high avidity may help exclude recent infection more reliably than low avidity can confirm it. Dziemian et al. found high-avidity antibodies in 94.2% of probable infections older than six months, whereas low avidity was present in only 25.9% of probable infections acquired within six months [35]. Avidity is also influenced by antigen composition, urea concentration, assay conditions, and the selected thresholds [12,36,37]. It should therefore be regarded as an additional marker of the probable timing and maturity of the antibody response.

### 4.3. Stray Dogs and Environmental Contamination Potential

The canine findings were high compared with most published estimates. A global meta-analysis reported a pooled prevalence of 11.1% in dogs, with higher values among stray and young animals [38]. In Astana, *Toxocara* spp. eggs were detected in 14.8% of 500 free-roaming dogs, while *Toxascaris leonina* eggs were found in 53.8% [39]. Russian studies have reported values ranging from 3% to 100%, with an average of approximately 33% [40].

A previous study in Uralsk detected anti-*T. canis* antibodies in 50.9% of stray dogs, eggs in 32.3%, and parasite DNA in 39.2% [41]. Although direct comparisons are affected by sampling and diagnostic methods, both studies indicate continued circulation of *T. canis* among stray dogs in the city.

Canine serology and copromicroscopy assess different aspects of *T. canis* infection. Copromicroscopy can detect patent intestinal infection by demonstrating egg shedding at the time of examination and is still a routine diagnostic method in veterinary practice. Serology detects antibody response after exposure but does not tell whether a dog has a patent infection or is currently shedding eggs. Maternally derived antibodies can also affect serological results in puppies. Dogs become infected by ingestion of embryonated eggs or larvae in paratenic hosts. Transplacental and, to a lesser extent, transmammary transmission are important routes of infection in puppies [20,21,42]. These routes help explain the high frequency of infection in young dogs and the continued circulation of the parasite in stray populations.

Once shed, *Toxocara* eggs require time to become infective but may persist in suitable environmental conditions. A global meta-analysis estimated that 21% of soil samples from public places contained *Toxocara* eggs [43]. Contamination has also been reported in public areas in Greece [44], urban parks in Valencia [45], and parks and soil samples across Latin America [46]. Studies combining human, canine, and environmental sampling have shown that prevalence in these three components does not need to be similar. In Brazilian communities, high human seropositivity occurred alongside lower canine egg detection and measurable soil contamination [27,28,47].

No soil, water, or other environmental samples were examined in Uralsk. Environmental contamination is therefore an inferred pathway based on canine egg shedding and established parasite biology, not a directly measured outcome. Future studies should include georeferenced sampling of parks, playgrounds, sandboxes, school grounds, courtyards, waste-disposal areas, and water sources. Molecular identification and assessment of egg development or viability would help determine where infective stages are present. Domestic dogs and cats should also be included because both *T. canis* and *T. cati* contribute to human exposure [19,21,29,48].

### 4.4. Experimental ELISA and Commercial-Assay Agreement

An experimental ELISA was performed using a gel-filtration fraction of a native somatic extract from adult *T. canis*. This antigen source was chosen because it could be prepared without egg embryonation or larval culture. The 18–22 kDa fraction was chosen because it gave the best discrimination between positive and negative sera, rather than the presence of previously identified proteins. Previous serological studies have also demonstrated the immunogenicity of adult *T. canis* extracts [49].

Humans are infected with migrating larvae, rather than adult worms, and hence larval excretory–secretory antigens are generally preferred for human diagnosis. Recombinant antigens may offer better standardization and specificity [31,32,50,51]. The native somatic fraction is easier to prepare and contains several antigenic components but may show batch variation and cross-reactivity to other helminths.

The experimental ELISA demonstrated good analytical separation, repeatability and correlation with the commercial DRG ELISA. Three discordant results were observed from 100 sera. However, the panel was chosen based on the experimental ELISA results, and no independent reference standard was used. Thus, the results indicate that the assays agree, but they do not provide estimates of diagnostic sensitivity or specificity. The assay should be considered as an experimental tool for the assessment of serological exposure rather than as a replacement for established clinical diagnostic methods.

### 4.5. Prevention, Study Limitations, and Implications

Control should focus primarily on definitive hosts and the environments they contaminate. Stray and domestic dogs require regular veterinary examination and appropriate deworming. Dog feces should be removed promptly and disposed of safely, and dogs should not be fed raw tissues that may contain larvae. Responsible ownership, registration, prevention of abandonment, and control of food and waste sources that support stray-dog populations are also important. These measures are consistent with the World Organisation for Animal Health approach to dog-population management [52].

Public-health measures should include hand hygiene, washing produce, safe handling of dog feces, and protection of playgrounds, sandboxes, school grounds, and water sources from dog access and contamination. Veterinary parasite-control recommendations similarly emphasize regular risk-based treatment, fecal management, and hygiene [53,54]. Coordination between public-health, veterinary, municipal, and environmental services is consistent with the Quadripartite One Health approach [18]. Human seropositivity should lead to clinical assessment only when supported by symptoms or relevant laboratory findings because IgG alone is not an indication for treatment [29,30].

The study has several limitations. It was cross-sectional; participants were volunteers; and questionnaire information was self-reported. Human clinical findings, eosinophil counts, and Western blot confirmation were not available. Canine serology may have been influenced by previous exposure or maternal antibodies, and age was estimated rather than known. Environmental contamination was not measured, and the use of an adult somatic antigen limits direct comparison with assays based on larval excretory–secretory antigens.

The main contribution of the study is the combined assessment of human exposure, IgG avidity, canine exposure, and canine egg shedding in the same urban setting. The findings identify stray dogs as an important target for control and provide a basis for future longitudinal and georeferenced monitoring that includes human, animal, and environmental samples [51,55,56].

## 5. Conclusions

This study provides integrated evidence that human exposure to *T. canis* occurs alongside a highly infected stray-dog population in Uralsk. The combined use of human serology and IgG avidity testing with paired canine serology and copromicroscopy allowed human exposure, canine exposure, and active egg shedding to be assessed within the same urban setting. The study also demonstrated close agreement between the experimental ELISA and the commercial assay in the selected serum panel. Although environmental contamination was not measured directly, the high frequency of patent infection in stray dogs identifies an important potential source of continued environmental contamination and human exposure. These findings support coordinated surveillance involving public health and veterinary services, regular deworming, humane management of stray-dog populations, prompt removal of dog feces, and protection of public spaces from contamination. Future One Health monitoring should incorporate direct environmental sampling to better define local transmission pathways and guide targeted control measures.

## Figures and Tables

**Table 1:** Interpretation of the anti-Toxocara IgG avidity index.

Avidity Index	Avidity Profile	Interpretation
<40%	Low	Less-mature antibody response
40–60%	Intermediate	Intermediate antibody-avidity profile
60–80%	Intermediate/high	Intermediate-to-high antibody-avidity profile
>80%	High	Mature high-avidity antibody response

**Table 2:** Final configuration of the experimental ELISA.

Parameter	Selected Condition
Antigen fraction	18–22 kDa native somatic fraction
Microplate	High-binding 96-well polystyrene plate
Antigen load	100 µg per plate
Antigen adsorption	24 h at 4–8 °C
Blocking	Not applied
Serum dilution	1:5
Anti-human IgG–HRP dilution	1:30,000
Goat anti-canine IgG-POD dilution	1:5000
Serum incubation	30 min at 37 °C
Conjugate incubation	30 min at 37 °C
Washing	Five cycles after each incubation
TMB development	15 min at 20–25 °C in the dark
Optical-density measurement	450 nm; reference 630 nm

**Table 3:** Reproducibility of the experimental ELISA.

Control	Intra-assay CV, %	Inter-assay CV, %
Negative control	5.45	1.80
Positive control	6.40	1.60

**Table 4:** IgG seropositivity to *T. canis* in the human study population.

Characteristic	Examined, *n*	IgG Positive, *n*	Positive, % (95% CI)	*p*-Value
Study year				<0.001
2025	500	85	17.0 (14.0–20.5)	
2026	500	31	6.2 (4.4–8.7)	
Sex				0.750
Women	729	86	11.8 (9.7–14.3)	
Men	271	30	11.1 (7.9–15.4)	
Age group				0.173
18–29 years	233	23	9.9 (6.7–14.4)	
30–44 years	298	29	9.7 (6.9–13.6)	
45–59 years	283	33	11.7 (8.4–15.9)	
60–74 years	175	29	16.6 (11.8–22.8)	
≥75 years	11	2	18.2 (5.1–47.7)	
Residence				0.741
Urban	905	104	11.5 (9.6–13.7)	
Rural	95	12	12.6 (7.4–20.8)	
Overall	1000	116	11.6 (9.8–13.7)	

Values are n/N (%) with Wilson 95% confidence intervals. Group *p*-values were calculated using Pearson’s χ^2^ test or Fisher’s exact test, as appropriate.

**Table 5:** Avidity profiles of IgG antibodies to *T. canis*.

Avidity Index	Avidity Profile	*n*	%
<40%	Low	33	28.4
40–60%	Intermediate	31	26.7
60–80%	Intermediate/high	34	29.3
>80%	High	18	15.5
Total		116	100.0

**Table 6:** Agreement between the experimental and DRG ELISAs.

Experimental ELISA Result	DRG Positive	DRG Negative	Total
Positive	47	3	50
Negative	0	50	50
Total	47	53	100

**Table 7:** Canine serological and copromicroscopic results by sex and estimated age.

Characteristic	Examined, *n*	IgG Positive, *n* (%)	Copromicroscopy Positive, *n* (%)
Sex			
Female	64	60 (93.8)	50 (78.1)
Male	36	33 (91.7)	28 (77.8)
Estimated age			
<1 year	24	23 (95.8)	21 (87.5)
1–3 years	46	43 (93.5)	36 (78.3)
>3–6 years	30	27 (90.0)	21 (70.0)
Overall	100	93 (93.0)	78 (78.0)

**Table 8:** Paired canine IgG and copromicroscopic findings.

Canine ELISA Result	Copromicroscopy Positive	Copromicroscopy Negative	Total
Positive	78	15	93
Negative	0	7	7
Total	78	22	100

## Data Availability

All data generated in this manuscript are available in the main text.
